# Interactions between youth and mental health professionals: The Youth Aware of Mental health (YAM) program experience

**DOI:** 10.1371/journal.pone.0191843

**Published:** 2018-02-08

**Authors:** Camilla Wasserman, Vita Postuvan, Dana Herta, Miriam Iosue, Peeter Värnik, Vladimir Carli

**Affiliations:** 1 Department of Child and Adolescent Psychiatry, New York State Psychiatric Institute, New York, United States of America; 2 National Centre for Suicide Research and Prevention of Mental Ill-Health (NASP), Karolinska Institute, Stockholm, Sweden; 3 Slovene Centre for Suicide Research, Andrej Marusic Institute, University of Primorska, Koper, Slovenia; 4 Medical Psychology Department, Iuliu Hatieganu University of Medicine and Pharmacy, Cluj-Napoca, Romania; 5 Department of Medicine and Health Sciences, University of Molise, Campobasso, Italy; 6 Estonian-Swedish Mental Health & Suicidology Institute, Tallinn, Estonia; 7 WHO Collaborating Centre for Research, Training and Methods Development at Karolinska Institutet, Stockholm, Sweden; Medical University of Vienna, AUSTRIA

## Abstract

**The Youth Aware of Mental health (YAM) experience:**

Youth stand at the core of much mental health promotion, yet little is written about their experiences of such efforts. We aimed to take this on by interviewing youth after they participated in Youth Aware of Mental Health (YAM), a universal mental health promotion program. YAM has a non-anticipatory methodology that provides youth with a safe space for reflection, role-play, and discussion. Addressing everyday mental health, YAM invites the experiences and issues relevant to the youth present to influence the program in a slightly different direction every time. The YAM instructor guides the participants but does not present the youth with given formulas on how to solve their problems. Like any mental health promotion, YAM appeals to some more than others in its intended audience and individuals engage with the program in many different ways. We set out to learn more about these experiences.

**Conversations about mental health:**

Thirty-two semi-structured interviews were conducted with 15–17 year olds in Estonia, Italy, Romania and Spain. In these interviews, the researchers made an effort to discuss mental health in terms relevant to youth. Still, wide-ranging levels of motivation, ease with engaging in dialogue with mental health professionals, and comfort with the format and content of YAM were detected. The youth were clustered in five different groups relating to their positioning vis-à-vis the researcher during the interview. The following evocative labels were used: “interested”, “foot in the door”, “respect for authority”, “careful”, and “not my topic”. Corresponding labels were devised for their YAM experience: “engaged”, “initially hesitant”, “cautious”, “eager to please”, or “disengaged”. We also observed that the researchers brought their own expectations and employed a variety of approaches that led to anticipating answers, stating the obvious, or getting along better with some of the youth. These modes of interaction were categorized under: “favoritism”, “familiarity”, “frustration”, “out of sync”, and “insecurity”. Similar power dynamics likely transpire in other encounters between youth and researchers, including interventions such as YAM.

**Youth and mental health professionals: Noticing the dynamics at play:**

As mental health professionals, we need to be aware of the professional habits and biases that sometimes obstruct us in understanding the experiences of youth. By initiating dialogue and listening closely to youth we can find a way to those experiences. Qualitative research can help bring the underlying interplay between mental health professionals and youth to the surface while also orienting the conversation towards topics that matter to youth. Some youth are more interested or feel more at ease in speaking openly with mental health professionals, while others find such exchanges less appealing or almost intolerable. Future mental health promotion initiatives would benefit from involving youth in the design of interventions to create an inclusive atmosphere and engage with topics that appeal to youth with diverse experiences of mental health.

## Introduction: Youth and mental health interventions

Remarkably little is written about youth encounters with mental health professionals and how these interactions may influence how youth experience mental health interventions. Even as they stand at the core of mental health promotion efforts and much published material, youth mental health is routinely addressed without engaging them directly.

The conception and design of interventions, as well as the interpretation and dissemination of study results, are often accomplished without input from the youth themselves [1, 2]. Furthermore, experiences pre- and post-intervention are typically evaluated through the lens of behavioral and health outcomes measured by administering standardized questionnaires that can provide an artificial sense of precision [3]. By relying on models for general behaviors, rather than an exploratory investigation of experience, we neglect to address youth beyond pre-existing psychologically-categorized groups [4].

The objectives of mental health promotion are numerous, but in no instance are the outcomes more important than youth being motivated and interested in participating. Our curiosity in youth experiences is lacking and as a consequence our understanding is faltering. Instead of entering the uncertain realm of assumption, engaging in dialogue and collaboration with youth is necessary. Moving beyond conceiving of youth as uniform entities and towards accepting youth as complex, fluid, and listened to by the research community, would help ensure the relevance of future mental health promotion [5]. Qualitative research can contribute to elucidate meanings attributed to behaviors and experience; yet such methods are rarely used in mental health research today [6, 7] and just as infrequently is such research accepted for publication [8].

An honest and open dialogue about the experiences, needs, and desires of youth is contingent on a critical perspective of the role of mental health researchers and the relationship between the two. As with any study population, but especially in the case of adults working with youth, the issue of power needs to be carefully examined [9]. Engaging in a more reflexive practice by assuming a critical approach to our position as mental health professionals can help us understand where current efforts are lacking or imperfect [2, 10]. Research and training that include consideration of the language, cultural orientation, and biases of professionals in mental health related fields remain rare [11].

### Youth mental health promotion initiatives in Europe

Two mental health promotion investigations preceded the current study: Saving and Empowering Young Lives in Europe (SEYLE) and Working in Europe to Stop Truancy Among Youth (WE-STAY) [12–14]. The SEYLE and WE-STAY Randomized Controlled Trials (RCTs) sought to assess school-based preventive interventions in youth [15]. Here, we will focus on one of these interventions, Youth Aware of Mental health (YAM), in greater detail [16].

#### Studying youth from the sideline

It has required several years to conceptualize, prepare, and implement the two aforementioned RCTs investigating youth mental health, and subsequently present the results in several publications [17–28]. However, throughout, the youth themselves remained remarkably absent in most of the research phases. Thousands of youth across Europe participated in these mental health promotion studies (12,395 in SEYLE and 11,186 in WE-STAY) and yet, their voices were missing from the process. Only after the completion of the RCTs, did we begin to seriously wonder about the YAM experience beyond a cause-effect paradigm. Prior to the current study, a qualitative effort to investigate the appeal of YAM among instructors implementing the program and lessons learned in the classrooms was carried out [29].

### YAM: Youth in play and dialogue

YAM is a program for 14–16 year olds that promotes increased knowledge about mental health. In the five-hour program spanning three weeks, everyday mental health topics such as peer support, stress, crisis, depression, suicide, and help-seeking are explored through role-play and discussions. Based on the premise that to combat mental health stigma, it is useful to start with “what matters most” to a particular group, the program relies on the input from the group of youth participating to influence the content [30]. The role-play activities are supported by an interactive talk given by the instructor, a booklet given to each participant, as well as posters that are hung on the walls of the classroom for the duration of the program. The YAM instructor, who does not know the youth ahead of time, sets the tone for a safe space and guides them through the topics while inviting their voices and experiences to remain central. In YAM, play offers the possibility to think about contradictions of the past, present and future in ways that allow for different forms of action [31]. No specific set of skills or rule-based discipline to promote mental health is offered [32]. The adult instructor does not tell the youth present how to solve their problems. Instead, reflection stands at the core as the youth contemplate how to care for themselves and support their peers by trying out and discussing different perspectives and experiences.

In the current study, we invited youth to speak about YAM and mental health. When analyzing the data, it became clear that understanding their YAM experience would not be possible without also investigating the interactions between youth and mental health professionals.

## Methods

### Study design and ethical approval

Qualitative interviews were conducted with thirty-two youth in Estonia, Italy, Romania and Spain who participated in YAM during the WE-STAY RCT. Sixteen females and males, respectively, between the ages of 15–17 years were randomly selected from the largest participating YAM schools in each country. This sample size was considered large enough to allow for variability in experience, yet small enough to feasibly conduct thorough interviews and extended content analysis [33, 34].

Ethical approval for the study was obtained from the European Commission and the participating country’s research ethics committee under the WE-STAY ethical approvals: National Institute for Health Development in Estonia; Comitato Bioetico de Ataneo of the Universita degli Studi del Molise in Italy; Comisa de Etica Universitatea de Medicina si Farmacie in Romania; Comité Etico de Investigacion Clinica Regional del Principado de Asturias in Spain. The WE-STAY protocol stated that the participating youth could be invited to take part in follow-up interviews. Parents or guardians signed written informed consent. Prior to giving oral assent, the youth were informed about the purpose of the interview and the confidentiality of the data collection and recording.

### Interview guide

Together Camilla Wasserman (CW) and Vita Postuvan (VP) designed a semi-structured interview informed by themes and queries that typically surface in YAM classrooms. To ensure comprehension and relevance to young people, a focus group with six 13–18 year olds was conducted, followed by a complementary informal conversation with a 16-year old girl. The focus group helped improve the format of the interview, including the order of the questions and to fine-tune the language used when speaking about topics like stress, bullying, and depression with youth.

We created an interview guide to enable us to compare styles between the two researchers carrying out the fieldwork. Directions included, for example, to not transform open-ended questions into closed-ended ones [35], to use clear and open language, and to be good listeners [36]. The researchers introduced themselves and encouraged the participants to explore YAM and mental health topics departing from questions prepared in advance. Following a few introductory sentences about the interview process, several concepts central to YAM were covered, including that there will be no right or wrong answers. Halfway through the interview, a mental health association game based on social representation theory was used to break up the conversation flow [37]. The game included 18 mental health related words, with the aim to explore meanings that the youth assigned to the words. Please see S1 File for a copy of the interview guide.

### Fieldwork

With local interpreters, VP led the interviews in schools in Estonia and Romania and CW in Italy and Spain. The interviews were mostly conducted in mixed languages: English and the local language. The interpreters were mental health researchers and professionals who collaborated with us in the WE-STAY study and here helped to carry out the interviews in the native tongue of the youth. The youth were free, and encouraged, to go back and forth between languages if they were comfortable enough in English to do so. The interviews lasted between 1.5 to 3 hours. The interpreter and researcher took field notes with observations about the youth, interview climate, room and general impressions after each interview.

### Transcription and translation

All interviews were transcribed verbatim and translated into English. To ensure accurate representation of the speaker, transcription guidelines prescribed keeping incorrect grammar and unusual word-usage, expressions, and sentence structures intact as well as to write out significant pauses, laughing, and interrupted speech.

### A note on language use

The researcher and the interpreter carrying out the fieldwork are both considered as *researchers* in our analysis as they played similar roles during the interviews. In the text they are referred to as *researchers* whereas in the quotes, in order to separate the three individuals present during the interviews, we write out both *interpreter* and *researcher*. In the text we sometimes refer to *professionals* in which case we denote both the researchers in this study and the instructors that delivered YAM.

All the citations from the interviews are quoted verbatim. As per the notation rules, (…), signifies a significant pause in speech.

### Data analysis

An Internet-based data analysis software for qualitative research called Dedoose [38] was used for data coding and allowed for simultaneous analysis by multiple researchers. Grounded theory was applied to explore the data and over time a two-fold analysis emerged based on the meaningful themes and topics identified [39]. The focus was initially on what the youth had to say about YAM and mental health, examining the interview as source [40]. However, over time, a reflexive analysis of the relationship between youth and researchers materialized [41].

The transcripts were coded “line by line” [39] and marked into units following shifts in the story line. In each unit, descriptive words, sentiments, or even whole sentences were identified by staying close to the words of the youth and researchers and not adding much analysis or interpretation. Next, commonalities were identified, and words and phrases were combined into higher order codes for the purpose of analysis [39]. Reflections not necessarily close to the text were kept as memos [3]. Throughout the analytical process, CW and VP discussed noteworthy themes from each interview to keep the narrative flow close at hand [42].

#### Transparency and thick description

In an effort to conduct the research as transparently as possible, close attention was paid to reliability criteria such as descriptions of the sampling, setting, and methods for data collection; identifying themes; and making use of supporting quotations [43–45]. The Results section comprises numerous extracts from the interviews in an attempt to include the actual voices of the youth not only while they participated in the interviews [46, 47].

To account for more than what was explicitly stated in the conversations between the researchers and the youth, we attempted to include some of the interview *texture* in the analysis. By texture, we refer to the broader context of the interview including the youth and researcher interactions and the youth responses beyond proof of opinion. Inspired by the anthropological practice of keeping detailed accounts of field experiences to help make social relations explicit, we conducted a *thick description* of the social relations between the researcher and youth. Thick description goes beyond a factual account of the research setting and includes interpretations and commentary in order to make the behavior or actions of an individual meaningful to an outsider [47, 48]. In the form of note-taking and discussions throughout the research process, attention was paid to the researchers’ values and experiences that played a role in creating the protocol, conducting the interviews, as well as interpreting the data [49].

## Results: Youth and researchers in conversation about mental health

Thirty-two youth were invited by mental health researchers to share their thoughts about YAM and mental health. Exchanges filled with feedback, both verbal and non-verbal, and interactions that ranged from difficult to fluid ensued. Here, we focus on the YAM experience by examining what transpired between the youth and researchers at the time of the interviews.

**Fig 1:**
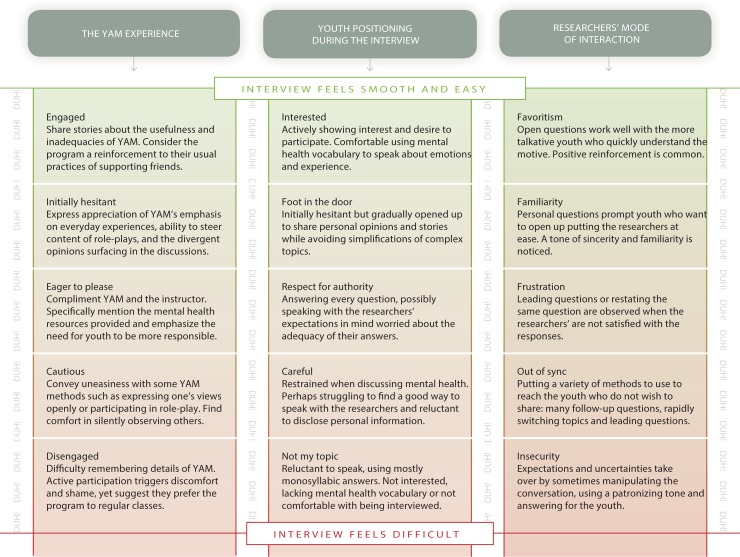
The YAM experience and the interplay of youth positioning and researchers’ mode of interaction. Fig 1 demonstrates the interplay between the YAM experiences (column 1) and the youth positioning (column 2) and researchers’ mode of interaction (column 3) during the interview. We observed a possible relationship between interactions between youth and mental health researchers and the adolescents’ YAM experiences.

Fig 1 demonstrates the interplay between the YAM experiences (column 1), the youth positioning during the interview (column 2), and the researchers’ mode of interaction (column 3). Attempting to unpack the YAM experience beyond what was explicitly stated by the youth, we observed a possible relationship between the adolescents’ styles of interaction with mental health researchers and their experience during YAM, as can be seen in column one and two. In the third column of Fig 1, we examine the researchers’ way of interacting with the youth during the interviews, which also relates to the two other two columns.

### The YAM experience

Below follows accounts of the YAM experience as outlined in the first column of Fig 1. Based on the adolescents’ feedback and analysis of YAM as well as what they remembered or did not remember of the program we consider their YAM experience. Five different categories of experience were observed, ranging from “engaged”, “initially hesitant”, “eager to please”, “cautious” to “disengaged”. Following the YAM experience, we move on to the interactions between youth and mental health researchers, also summarized in Fig 1.

**Engaged: Recognizing emotions. N: Four females and three males:** The youth referred to as “engaged” voiced an interest in mental health and psychology. They had previously contemplated mental health topics, both in relation to self and others, and told us that they “talked to their friends about these things [mental health, emotions etc.],” while sprinkling their speech with YAM buzzwords like stress, coping, support and communication.

*Girl*: I tend to leave everything for the last minute

*Researcher*: Ok

*Girl*: And that doesn’t mean I can’t cope in that condition, when I’m stressed I can do miracles (…)

*Researcher*: Ok

*Girl*: But that’s not what I want and how I want to feel.

They found the program to be beneficial, specifically pointing to the topics tackled and the methodology used:

*Researcher*: And, so, overall, how did you find the program?

*Boy*: Really good. I’ll say it again, I liked it. I liked what it dealt with and the fact that it was, well, (…) dynamic, you know what I mean? Explaining these type of things a bit. I liked the fact that, that at this age it made people think about problems of that type, you know what I mean? That if something’s happening to you or to someone who is close to you, they would look on it positively, value it, give it importance.

Many of them told us that they help their friends when in need and that they appreciated YAM as reinforcement. As one girl put it:

I mean, it’s not so rare to suffer from depression at this age when your life is changing (…) a lot of things are changing, and that can make you think about things more (…) clearly. It’s important for there to be a point of support, eh? Or these types of talks, so that you don’t feel totally marginalized if these sorts of things happen to you.

Debates and noteworthy role-plays were recounted, sometimes recalling conflicting ideas that surfaced regarding how to solve a problem or approach mental ill-health. When giving their opinions on YAM, they would break down the program, telling us what they liked and didn’t like, or how the program could be useful for different kinds of peers that they identified. As one girl told us:

It’s good to have these booklets, because you can read them too, but I think it’s good to talk also. It’s very important that you can and know how to talk, because everyone knows to ask, but nobody knows (…) I mean nobody (…) lots of people don’t know how to say something like ah to express themselves in a better way or to try to communicate, they just talk (…) it’s hard to find your voice to fully express them [emotions]. I think that.

While another girl explained:

*Girl*: One way or another this type of program can help you. Whether it’s you helping someone or someone helping you. And it seems that, yes, everybody can manage to understand it, to realize that these are real problems, especially in this age range, and I think it’s relevant for all types of people.

*Researcher*: But you mentioned there were people in class who were a bit immature. For them, for example, can this type of program help them?

*Girl*: Yes, I think that in some way, taking stock, not of other peoples’ situations, but your own, I think it can help you to, like, look at yourself anew. Maybe it’s worth it when things weigh you down so much, knowing that sometimes it can turn into depression, or whether it’s better for you to try and make your character stronger, no? In response to the things that happen to you, in response to life. I think these programs are relevant for everybody.

They recalled participating in the role-plays, maybe even being the first to volunteer, like this girl:

Usually we are really really shy, so we’re not like “pick us, pick us”, I’m the only one crazy enough to say: “me, me, me, I don’t know what it’s about but pick me!”

Speaking about help seeking, they often gave nuanced analyses. Here, a boy talks about the mental health resources listed in the booklet and on the posters that all participants received.

I think it depends a bit on the person, because, I mean, some prefer having someone who is a bit closer, someone they can talk to, because it’s someone who is closer to them, and they prefer that to dealing with someone who is official. Because maybe that person makes them feel less safe. But I think there are also people who may prefer more confidentiality and would rather talk to someone who is official, and maybe more formal. Yes, I think it depends on the person. Whatever you feel more comfortable with.

**Initially hesitant: Life experiences. N: Four females and four males:** Particularly interested in life experiences, their own and others, a girl here reflected on what YAM meant to her.

*Researcher*: So, you remember a bit, it was called an awareness program. I don’t know if you recall? And what does that mean to you?

*Girl*: Well, hm (…) To raise awareness in ourselves about the problems that are out there, not just in our lives, but that there are also other people suffering in ways that we maybe don’t suffer, but, we can also help them to get out of it.

These adolescents may not always be immediately recognizable as actively participating in YAM because they were not always motivated to speak from the outset, which is why we grouped them under “initially hesitant”. However, when they saw an opportunity to express personal experience and opinion, they did. It is possible that what played out in the interviews also occurred during YAM; a slow warm-up was necessary before common ground was found. One boy told us that he didn’t pay much attention at the time of the program.

Because I was listening with one ear only and in the other one I had headphones! ((laughing))

Nonetheless, he subsequently spoke in great detail about YAM topics that he remembered and that mattered to him, namely marijuana, about which he had many opinions and some experience.

YAM sets the stage for discussion by inviting youth to bring up topics and without providing them with a set formula on how to solve quandaries that surface in everyday situations. This group of youth found the personal interview questions and YAM content generated by their own experiences particularly appealing. The use of a more open-ended approach invited multiple take-aways. Bringing this reflective aspect of YAM to light, here a boy emphasizes two key take-aways from YAM: the importance of considering the consequences of one’s actions, and the idea that every situation can look quite different depending on who is involved.

You’re showing people what can happen in cases of bullying, like, seeing what can happen to you if you’re involved in a problem like that. Seeing the problems it brings and the consequences it has for you, when you’re bullying someone or when you’re being bullied, or they’re doing whatever to you.

Reflecting back on YAM and the period after the program, one girl said:

I remember that it was somehow (…) maybe it made our class friendlier if it was this study.

Some expressed that they weren’t interested in “cheesy self-help talk”, showing sensitivity to how mental health is presented. Typically, when the youth had an experience related to a particular subject matter, they recalled the role-plays from YAM that touched on it and told us anecdotes about the performance and the person who had role-played. Memorable role-play sessions also included funny situations, such as switched genders or someone pretending to be an adult.

No, the topics were examined in-depth, but I felt like laughing (…) they were (…) it was fun.

Recalling specifically heated debates, they mentioned classmates voicing opinions or sharing experiences they previously did not know about. One girl spoke to us about diverging opinions in relation to emotions:

Well I mean there are people and people. There are people who don’t have feelings, and they don’t care what’s happening, because they don’t even care what’s happening to themselves, so (…) there are people who aren’t interested. But there are also people who are indeed interested, because they’ve seen situations that didn’t seem fair to them.

Some said it was impossible to feel bad coming to school if you were being bullied. And others among us said yes, you were bound to feel bad. Seeing that person who bullies you every day and suffer. So yes there was some debate.

The real life applicability of YAM was also discussed, such as how to help a friend who was going through a difficult time after the program had ended. This girl told us:

Yes, for example, a friend whose parents had problems and split up, and she went through a terrible time, because her parents left, and she had it rough because her dad went to live abroad and her mother stayed here. So, it was a question of how to help her, because she was closed in on herself, and didn’t want to do anything, so it was a case of, like, how to apply it to situations like that.

Youth in every group told us they had the booklets tucked away somewhere at home, maybe in a drawer. In this group some showed more interest, mentioning that they had looked at the booklet again, like this boy:

Hmm (…) a few times I think when I was cleaning the room and it came to my hand.

Another boy spoke about the list of community resources, pointing out that he had not known about the mental health ones previously:

*Researcher*: And was the list useful?

*Boy*: I mean there were some I already knew like for example the emergency and civil defense and things that we knew, but for example the mental health center and that I didn’t have [before].

**Eager to please: Positive feedback. N: Four females and two males:** While many of the youth interviewed did not immediately remember YAM or exactly what they had done in the program, the “eager to please” group made considerable effort to do so. They enumerated the different program components and complimented us on the program. In conversation, some of them marked a certain distance from others their age, lumping youth together, perhaps with the intention of coming across as more mature. This girl told us:

No no no, I remember. Oh well, during the project, last year, we had several meetings and in these meetings we also did some games useful for youth. However, it was also very interesting because it also made us, I mean, it made us understand the problems that we as adolescents (…) we have today.

They often spoke highly of the YAM instructors, particularly with respect to keeping everyone interested and calm. One girl said:

There was never a situation in which [the instructor] had to become angry because a kid made noise (…) it never happened.

Or in this case, describing how the class respected the YAM instructors:

*Boy*: No, because people from outside come, and they’ll always be respected more than the teacher.

*Researcher*: Oh yeah? Respected?

*Boy*: Always, because he comes to do an activity and you’re more (…) I dunno (…) you pay more attention to what he does, and that.

If the instructors put them to relative ease, then at times the presence of their peers did not. Simultaneously voicing some reservations in regard to the potential judgment of her classmates, yet wanting to give positive feedback to the researcher, this girl spoke about how comfortable the program made her feel.

Eh well, at the beginning however you are with people who, I mean you don’t know them, you don’t have a strong relationship or friendship (…) you feel a little uncomfortable. You feel like you’re being observed (…) but at the end it’s fun. It’s also nice because then it (…) maybe because I was with good kids who were nice, but anyway they can judge you. I mean I felt very comfortable.

When analyzing the YAM experience, most of the youth in this group gave rather general statements about what they had learned in the five sessions. The following girl told us that the role-play situations were fun, broadly relatable, and that she felt the urge to be more responsible now, tending towards a desire for maturity and independence.

*Girl*: Because like these are situations that can happen to everyone so at the end the question comes to mind, what would I do. It was fun yeah!

*Interpreter*: Mmm

*Researcher*: And did you learn anything?

*Girl*: Mmm I feel like I’m, like I’m a bit more responsible (…) than before. I don’t know why but I feel maybe a bit more responsible, more responsible, yeah.

They drew attention to the list with mental health and community based resources, perhaps accentuating their respect for professionals. One boy said:

They also gave us the email of the psychologist in case, in order to (…) if we wanted to contact her to talk about our problems.

Notwithstanding their appreciation of mental health professionals, several youth in this group mentioned that one should take care of oneself and not rely on others. This girl expressed the need to pick yourself up and move on.

It’s all about the way you think and the way you want to think. Usually if you want to feel sick you’ll really feel sick. Even if I was sick and I had some pretty severe health problems, I managed to recover thinking that I will be all right. I don’t want to stay all day long thinking about my problems, I think it’s very important to know how to educate yourself.

A sense of not just taking care of yourself, but the necessity to not focus too much on mental ill-health was also voiced. In YAM, although it is up to the youth to find the best ways to care for themselves and their peers, support from others is always encouraged. Few youth in this group appeared to embrace this sentiment. We asked what kind of program the youth would want to create themselves to help others and one girl told us:

I would make them grow up

**Cautious: Not in front of others. N: Four females and one male:** Cautious to not share too much, this apprehension affected these adolescents’ YAM experience. One boy said that he was not comfortable in expressing his views to the whole class:

In public you are even less likely to tell the truth than maybe in the questionnaire (…) for me, in the questionnaire, at least, that‘s true, for example I’ve got five brothers and sisters–that’s true–but when I have to say it in public maybe I say I have just one (…) What does it matter to people how many I have? I think the truth comes out in writing even more.

Inquisitiveness or concern about the anonymity of the study may have influenced how some of them responded to YAM.

*Interpreter*: Was there anything in the program you didn’t understand? Anything you remember, like…?

*Girl*: The only thing was the way it was kept anonymous, how it was done, with the stickers. That was a bit complicated, actually.

Although they recalled funny or unique role-plays, they did not take active part in the enactments. One girl told us she specifically appreciated the role-plays because she enjoys observing at a distance.

I like to imitate people. I mean, like, when I look at somebody I notice all the details: glasses, everything, everything.

To others, the very methods of the role-play and discussions were problematic.

*Boy*: Specifically, for example, the role-playing I mentioned, it’s more of a group thing. So it’s more complicated, I mean, when you’re on your own.

*Interpreter*: Do you prefer things you can do alone?

*Boy*: Yes, simpler

*Researcher*: And (…) simple, in which way? I don’t really understand that.

*Boy*: When you do something just for yourself, it’s simpler to know what you’re thinking, than it is with a whole group, with lots of people. It’s harder to get everyone to agree when there’s a lot of people than when it’s just you.

The “cautious” group did not necessarily actively participate in the discussions or remember whether or not they did. Leafing through the booklet, one girl told us:

*Interpreter*: So what are you thinking when you’re looking at this?

*Girl*: Well, about how I would have reacted. And, problably, I agreed with many things, with some of the reactions and I maybe did make some comments, a little bit, on (…) if I, if I (…) I think I did say something.

YAM invites the discussion of different outcomes in dilemma like situations or pertaining to everyday mental health problems, however not everyone appreciated the divergence of opinions this brings. To some, the hands-off style of the instructor caused some confusion and disapproval. This boy told us:

He was just doing his job, he didn’t care if someone talked or another did something else. I don’t know why he did that (…)

At times the content of some of the YAM materials appeared confusing or misleading. Here a girl describes how the description of depression presented in the materials was not to her liking.

*Girl*: Ok, for example, the thing about depression. As far as tiredness goes, I’m a really lazy person, who’s tired a lot of the time. But I’m not depressed, I don’t have any problems in that regard. Things like that.

*Researcher*: But did the instructor explain that these are things that happen to a lot of people, but that, for example, it’s not always just one [symptom]. Or did they not explain it well?

*Girl*: No, I mean, yes, afterwards when we were talking and reading about it, yes, they told us that obviously, if it was just one of these things, it didn’t mean you were suffering from something, but in any case these are things that (…) if someone wasn’t there when they did the talk, say, they might read those [booklet and posters] and come to the wrong conclusion.

In her critique, this girl actually points to one of the key components of YAM, consideration of the often contradictory and complex expressions of mental health. Yet, it appears, exploring together as a group did not serve the same function for everyone, as some youth looked to the written materials for answers. When prompted to change something in the program, one boy told us he would add “stories of people who lived actual stuff”.

**Disengaged: Indifferent perhaps. N: Six males:** During the analytical process, we initially had difficulty noticing these individuals, mainly because they did not have much to say about YAM. However, the coding of long silences, prompting or leading questions, as well as many “yes” and “no” answers tell a story beyond verbal clues. Their comments about YAM chiefly concerned whether or not they remembered the program. They were clearly “disengaged”.

*Interpreter*: Do you remember that there were some workshops, role-plays?

*Boy*: Aaah (…) no.

*Researcher*: It’s normal, it’s normal ((laughing))

*Boy*: No, I wasn’t so curious to know what was going on there, what’s with this project.

Some appeared pressured by our questions about program details, such as what they remembered or how many classes they attended.

I don’t know what to say to you, I mean, since I don’t usually go to many of the class tutor sessions, I don’t know what to say to you. But the booklet, yes, I was in that class at least, but it’s not from last year, eh. Or did you give them out last year?

If they did remember, they stated whether the activities were fun or not. There was not a lot of motivation to participate in YAM and they reported taking part because others did or because they preferred it to regular classes.

*Researcher*: Do you remember that maybe you had to do role-plays or workshops?

*Boy*: No.

*Researcher*: No? Or maybe you remember this booklet?

*Boy*: I didn’t see this book.

*Researcher*: ((laughing)) Maybe they didn’t give it to you.

*Boy*: I think that they gave me this book but.

*Researcher*: Have a look, maybe?

*Boy*: Maybe I took this book but did I read it?

*Researcher*: ((laughing)) Now we did some interviews and we want to see if people remember, but you don’t remember at all? No?

*Boy*: No.

*Researcher*: What about the topics? Do you remember that you discussed these things?

*Boy*: I remember that this is the topic of this thing actually, everything that’s here [in the booklet].

*Researcher*: Yeah ok, so nothing else comes to mind?

*Boy*: No.

Many were reluctant to express themselves on YAM topics, rarely using explicit mental health vocabulary. An example of this is demonstrated above when the boy above speaks in a roundabout way: “this is the topic of the thing”. In addition to simple monosyllabic answers, they would say things like “I don’t know”, “I never experienced anything like that”, “that’s all”, and as described later, these responses activated insecurities in the researchers. One boy put it in a very frank manner when asked about the mental health related words of the association game:

*Boy*: Hmm ((laughing)) ahh (…)

*Researcher*: Are these hard words, difficult words?

*Boy*: Yes

*Researcher*: Why are they difficult?

*Boy*: I don’t know ((laughing)) I don’t know what to do with these words.

Some of them brought up feeling uncomfortable or too ashamed to actively participate:

*Boy*: We played some scenes.

*Interpreter*: Mm ah scenes. Did you take part in them or?

*Boy*: No!

*Interpreter*: Did you just watch? ((laughing))

*Researcher*: Why? ((laughing))

*Boy*: No, I’m too ashamed to do that.

Another told us:

Because acting things out like that (…) It’s just a bit of a laugh–it doesn’t serve any other purpose. You don’t learn anything from it, that’s what I think, eh (…) just my opinion. I don’t think it’s good for nothing.

Others did not see the benefit of discussing mental health in the open and doubted it would lead to any outcome of interest. Focused on changing behaviors and how to solve problems, this boy critiqued YAM for being directed at older teens and wondered whether it is possible to influence anyone’s behavior at all.

I know people in sixth grade smoking so I think action should be taken from a young age. If you are in the twelfth grade at eighteen no one can change us anymore. This is my opinion.

I don’t know if these problems can be solved, I really don’t know. It’s up to the person if he chooses to do right or wrong.

Some described shame connected with voicing ones opinions or experiences. YAM was no exception. Here on the topic of bullying and YAM a boy tells us:

*Boy*: I mean, they’re private things, I don’t think even your best friend tells you those things, I just don’t think they get talked about. In class I don’t say anything, and nothing happens to me, and nothing happened to me, they don’t bully me, so.

*Researcher*: But, for a person who’s being bullied or abused, could it (…) could it be useful?

*Boy*: Well, I don’t know if they dare to confess something or whatever, maybe yes.

While speaking about the mental health resources provided in the YAM booklet, the conversation wandered to whether one should seek help from professionals at all. This boy underscored the importance of staying out of other peoples’ business.

*Interpreter*: If you remember there was a list of contact people there?

*Boy*: No no.

*Interpreter*: Would you ever contact these people?

*Boy*: No no.

*Interpreter*: Why not?

*Boy*: Well, I didn’t have (…) why…

*Researcher*: What if you would have some problems, would you contact?

*Boy*: If I had had a problem?

*Interpreter*: If you had a problem.

*Boy*: Yes.

*Interpreter*: Do you know someone that has problems and they should (…)

*Boy*: No.

*Interpreter*: (…) should contact those people?

*Boy*: Don’t know. Each one, they don’t say whom and what (…) to each their own.

### Youth positioning during the interviews

Positioning here refers to the youths’ inclination to speak about YAM and mental health topics as well as their comfort level interacting with the researchers at the time of the interview. The positioning was not necessarily expressed explicitly during the interview but revealed in the analysis. Though their responses ranged from puzzled at our interest in their YAM experience to immediately comfortable speaking about mental health, the youth were never completely silent, but were sometimes reserved. Throughout, the researchers tried to refrain from dominating the conversations by inviting the youth to set the tone with their choice of language, the amount of silences required, the desire to go deeper, or to terminate any given conversation. As is the nature of semi-structured interviews, each exchange was dissimilar from the last, yet the youth tended towards one of the five positions that can be found in the middle column of Fig 1. The descriptive labels for the five groups correspond to those described earlier in the YAM experience section (“engaged”, “initially hesitant”, “eager to please”, “cautious”, and “disengaged”).

**“Interested”: I know how to do this.** In these interviews everyone appeared to be at ease, some explicitly expressing that they liked talking to us. For example, one girl told us:

I liked it. It’s good, the questionnaire and discussions [in YAM] and this interview and everything.

The youth were “interested”, some even expressed it as such:

*Researcher*: How was it now talking to us?

*Girl*: Interesting. Very interesting.

*Researcher*: Yeah?

*Girl*: Yes, and I enjoyed doing it.

The participants opened up, sometimes beyond our expectations, clearly having the experience and vocabulary to do so.

*Researcher*: What would they say about depression?

*Boy*: Hmm, yes, I do remember (…) a bit about the symptoms, like, why you can get to that point (…) and I remember they also said it could be because of the parents, because maybe they ask too much of you, and maybe they force you to do things you don’t want to do, so that you can reach a point where you like, I mean (…), you just can’t stand it any longer and you’re overloaded.

By using language and referring to content familiar to the mental health professionals interviewing them, they kept the conversation going without much probing.

**“Foot in the door”: Give me a little time to speak the way I like.** Categorized under “foot in the door”, these individuals were initially reluctant, but gradually opened up over the course of the interview as a tone of familiarity developed. Telling us what he thought when asked to participate in the interview, one boy said:

*Boy*: Two of my classmates, who were called together with me said, “Do you want to do it?” one said: "I don’t know, maybe yes", so |then] they said: "You do it as well, because we will do it too" and I said "Oh well, I want to do it too!"

((Everyone laughing))

*Researcher*: And how did it feel? In the end?

*Boy*: Well it was pleasant (…) then and in- yes interesting and also useful.

*Interpreter*: Useful, useful how?

*Boy*: Useful as all the things that you did with us! ((laughing))

Some seemed particularly indifferent to topics that bordered on generalities, giving short answers to such questions. On the other hand, more personal or open-ended questions stimulated the sharing of stories.

Girl: I liked it because there are always things that, I mean, there are always things that help you to understand, to grow.

One boy expected to mainly give us feedback on YAM. When the interview was over, after instead having spoken about more personal issues, he told us:

*Interpreter*: How do you feel now? Or what did you think before you came here?

*Boy*: No really, also before coming here I was (…) I didn’t feel anything special, I didn’t know, well what (…) I thought that they were questions about (…) I didn’t think it was this, I only thought it was about the beginning when we talked about the meetings in the past year

*Interpreter*: Ah, the past year

*Boy*: I didn’t think it was about all these things. But, I’m feeling, I’m feeling good, because I talked, I opened up about myself.

**“Respect for authority”: Am I doing alright?** Some of the youth, whether talkative or not, seemed to answer questions with the researchers’ expectations in mind. Hence, we grouped them under “respect for authority”. The youth answered our questions without difficulty, but at the end of the interview we had the feeling that we did not get any closer to them in conversation. Speaking because they were expected to, perhaps to make a good impression, and feeling pressed to answer every question, some even thanked us for coming.

*Girl*: Well, I think that we honestly got wiser. And thank you very much that you took the time and came here.

At times they seemed unsure of how to answer and uneasy that they could not do so in a way they deemed adequate, afraid of not having the right answers. When asked how they felt about the interview one girl told us:

Actually at the beginning I was a little bit nervous because I did not know what to say. And now what will they ask me? But everything went well ((laughing)).

Some expressed that they were happy and surprised to have been invited to participate.

*Boy*: I just thought what are the odds of me being chosen from those thousands of students.

**“Careful”: Can I trust you?** Though many of the interviewed youth were not immediately comfortable speaking with us, certain remained reserved throughout. Perhaps they were unwilling to share their thoughts or not able to identify an acceptable way to speak with us.

*Researcher*: How did you find this interview?

*Girl*: At first I was very nervous. But anyway, I get very nervous about stupid things.

*Researcher*: And now?

*Girl*: Now I’m feeling calmer.

Revealing very little about their private lives, but still mindful to answer all of the questions albeit without disclosing personal details, we referred to these youth as “careful”. A reoccurring inquiry from this group was whether they should give personal or general responses.

When asked which parts of the interview were more challenging, one girl unambiguously pointed to subjects bordering on the personal.

Maybe, for example if you ask about specific problems I might have at home, or with my friends, (…) more personal things, that might create more problems, but as for the rest of it [it’s ok].

Some specifically told us they prefered topics that were not about feelings.

*Interpreter*: Which was harder, to talk about your feelings or when you talked about skipping school?

*Boy*: No. About feelings.

**“Not my topic”: Let silence speak.** With the agenda of mental health set, some of the youth found it difficult or undesirable to actively participate. Grouped under “not my topic”, they were not interested in speaking about emotions, expressed indifference and were sometimes critical. These youth were not very talkative and the interviews were often short.

*Interpreter*: How did you feel during the interview?

*Boy*: Ok.

Many were not fluent in mental health vocabulary or had little relatable personal experience. During the mental health association game, many of them struggled with some of the more difficult words.

*Interpreter*: Self-esteem.

*Boy*: That one, what can I say? Self-esteem is if each person (…) what does self-esteem mean?

Some were critical of the evaluation questionnaire and YAM program, which informed their impression of the interview, as expressed here when we asked what this boy thought about the interview:

*Boy*: I understood that you wanted to do this because you wanted to do a more focused study.

*Interpreter*: No, to find out a bit about the students’ opinions.

*Boy*: Better because at least now you have sincere opinions, because otherwise there’s no sincerity in this [evaluation questionnaire and YAM]. And apart from that, nothing.

### The researchers’ mode of interaction during the interviews

Observations regarding the researchers’ interview methods and techniques can be found in the rightmost column of Fig 1. Following the interview protocol, the researchers initiated every interview in the same way. To start off, attempting to set a relaxed tone, they chatted informally, introducing themselves, letting the participants know that there were no right or wrong answers while assuring the confidentiality of the conversation. However, as is the nature of semi-structured interviews, the dynamic of the ensuing conversations varied. The researchers provided guidance, through questions and sympathetic conversation prompts, to ensure that the interactions were cohesive and intelligible while following the thread of the interview topics. They also turned to the youth to provide topics and steer the interactions. Through this method, the resulting content of each encounter was unique.

#### Navigating from smooth to difficult

Similar to the five positions we observed with the youth during the interviews, we also identified five tendencies or what we refer to as mode of interaction used to conduct the interview. It is of note that these different approaches were not immediately observed at the time of the interview, as the researchers themselves were involved in the dynamic. Rather, these modes of interaction were identified in the analysis.

#### Favoritism

Favoritism on the part of the researchers was noted in the conversations with youth who spoke with fluency and ease about mental health. These interviews provided a sense of relief from others that were more difficult to conduct. The more difficult interviews required a great deal of effort from the researchers to facilitate a response from the youth. However, when the youth were more outgoing, the researchers were more likely to offer open-ended questions. The “interested” youth would even interrupt the interviewer to respond. As a result, the researchers’ questions were typically shorter than the informative answers provided by the youth.

*Researcher*: And these issues, had you thought about them before this program?

*Boy*: Well, I’d seen it on the news and stuff, cases of boys and girls who well, committed suicide or something. There was that case in the media of a girl who showed her breasts on the Internet and then was bullied on Facebook and social networks, and eventually committed suicide. She uploaded a video onto YouTube and then killed herself. I don’t remember her name.

*Researcher*: Here?

*Boy*: No, in the United States.

We observed that there was a tendency for the researcher to provide positive reinforcement when the youth gave nuanced feedback about YAM. For example, at the end of the interview, heartfelt thank-yous were exchanged:

*Interpreter*: Super, thank you actually!

*Boy*: Thank you too!

*Interpreter*: Well, this is like a work and fun at the same time, big thanks to you for coming here.

#### Familiarity

Youth in the “foot in the door” group were particularly keen to speak about their mental health related experiences. The researchers often seized on the opportunity to ask personal questions, leading to sincere exchanges with a tone of familiarity.

*Interpreter*: So, do you think that a person can be helped with these problems [in reference to the words in the association game]? Can they do something?

*Boy*: Of course. A person must be helped and must do something for himself. Like getting help or facing his problems.

*Researcher*: Do you know any example of someone who was helped?

*Boy*: When I attended junior high, a girl was depressed and she didn’t want to live anymore because she was rejected by both the boy she used to date and other friends too and she was depressed, she had not (…) she had no reason to live, so she started crying, she said she wanted to die, so in that moment some teachers helped her and they also had (…) there was also some improvement in the class, to help her.

Researchers felt at ease once the youth opened up, the conversation was typically relaxed and peppered with witticisms. Here a girl speaks about her peers humorously, making the researcher laugh and bringing about a continued tone of proximity.

*Girl*: Some people are weird, they start drinking in the morning and then get sober for the evening so they can go back home.

*Girl and Interpreter*: ((laughing))

#### Frustration

Interviewing the “respect for authority” group sometimes required the researchers to add additional explanations to clarify what was being said. Conceivably due to frustration of not getting what they wanted from the responses, the researchers often turned to leading questions or restating the same question in different ways. For example, when asking about the YAM instructor:

*Interpreter*: How was she? What impression (…) what would you say?

*Girl*: No, no, she is nice, she is good. No, no, a good person.

Researcher: And was it (…) did she explain things to you in a good way or?

*Interpreter*: Do you think she explained the things well or?

*Girl*: Yes yes

*Interpreter*: Did she make herself understood?

*Girl*: She explained all the details to us

The unintentional effect was that the additional explanations may have hindered the youth from speaking or using their own words. Sometimes this was due to translation issues or trouble understanding the question, but it was also a result of the researchers trying different means at their disposal to get more out of the conversation.

*Interpreter*: Do young people have these problems that were mentioned there like depression and stress and bullying?

*Girl*: Maybe. Yes.

*Researcher*: How does this look like in young people? We are a little bit old, we don’t remember anymore, so you have to tell us.

*Girl*: I don’t know the problems of today. There is bullying. For example I think too much so this is going under emotions. Well (…) I don’t know.

*Interpreter*: There is definitely bullying but if she thinks about herself then you can overthink.

*Researcher*: Overthink?

*Interpreter*: And then you start to get problems.

*Girl*: Yes.

*Researcher*: What does it mean, overthink?

*Girl*: For example if (…) ohh I can’t explain.

*Interpreter*: Well, if problem seems bigger than it is.

*Girl*: Yes, exactly.

The researcher adding, “we are a little bit old” could be in response to sensing how the youth in this group sometimes distanced themselves from their peers. This may have been an attempt by the researcher get closer to the youth.

#### Out of sync

In some instances the researchers appeared out of sync with the youth, particularly those who were reserved or apprehensive of YAM in the “careful” group. The researchers were scrambling to reach the interviewees who did not immediately share. By using numerous follow-up questions to attempt to make a connection, they sometimes ended up switching the subject too rapidly and not waiting for the youth to respond.

*Researcher*: No, that was ok. So you liked that or you didn’t like this questionnaire?

*Boy*: Hmm. Can’t complain.

*Researcher*: Can’t complain ((laughing))? Do you also remember that you did a workshop? Some role-plays?

*Boy*: No.

*Researcher*: No, nothing to remember. Or did you have a lecture? Somebody tell you something about maybe stress? (…) No, I am just asking. If you remember, then say yes.

*Boy*: No, I don’t.

*Researcher*: You don’t.

*Interpreter*: Role-plays were done to talk about some problem and you were asked to act a certain role, for example one person who bullies and the other who is bullied. These games were something like that.

*Boy*: (…) No.

*Researcher*: No ((laughing))

*Boy*: I don’t have the best memory.

On and off in the interviews, the adults were clearly dominating the dialogue as seen above. Here in a conversation about the role-plays, one of the interpreters asks particularly leading questions to a girl in the “careful” group:

*Girl*: Eh I mean (…) it was nice, I mean, they felt comfortable, because it was interesting.

*Interpreter*: A little bit embarrassed or not?

*Girl*: Eh, at the beginning no one wanted to do it, but at the end (…) we did them.

*Interpreter*: ((laughing))

#### Insecurity

In the particularly difficult interviews to conduct, when the youth had little to say, the researchers’ insecurities often took over. When they were not satisfied with an answer, they sometimes became pushy. For example, in a quotation found earlier under the “Not my topic” category, the researcher manipulated the conversation by employing a patronizing tone and even appeared to laugh at the boy. Let us revisit the same extract from the interview here.

*Researcher*: Do you remember that maybe you had to do role-plays or workshops?

*Boy*: No.

*Researcher*: No? Or maybe you remember this booklet?

*Boy*: I didn’t see this book.

*Researcher*: ((laughing)) Maybe they didn’t give it to you.

*Boy*: I think that they gave me this book but.

*Researcher*: Have a look, maybe?

*Boy*: Maybe I took this book but did I read it?

*Researcher*: ((laughing)) Now we did some interviews and we want to see if people remember, but you don’t remember at all? No?

*Boy*: No.

*Researcher*: What about the topics? Do you remember that you discussed these things?

*Boy*: I remember that this is the topic of this thing actually, everything that’s here [in the booklet].

*Researcher*: Yeah ok, so nothing else comes to mind?

*Boy*: No.

The researcher underscored that the boy does not remember aspects of the YAM program and even uses it against him. Other times, the researchers were able to restrain themselves at the last minute from answering for the youth being interviewed. An example of this occurs at the end of this short dialogue:

*Researcher*: Right, in general, is it OK to skip classes or is it a bad thing, in your opinion?

*Boy*: Neither good nor bad. Each person decides (…) I don’t know, if you’re bored you leave, and if not, you don’t.

*Researcher*: So, in life, if something bores you (…)

*Boy*: You drop it.

*Researcher*: You have to change the situation.

*Boy*: I think so.

*Researcher*: So, according to you, the reason people skip school is (…) or what are the different reasons?

### Dialogue despite previously formulated definitions

The language that researchers use in their professional lives is strongly influenced by commonly-used psychiatric terminology. This way of speaking sometimes materialized during the interviews and would sometimes escalate when unfamiliar definitions were not understood or accepted, at times even resulting in a disagreement with the youth. When the youth had little to say, the researchers often interjected. Below, a boy does not know what to say about depression during the association game, provoking a condescending reaction:

*Boy*: Difficult one (…) not sure what to say (…) it’s really difficult. Don’t know what to say about depression.

*Interpreter*: Why is it so difficult?

*Boy*: I don't know (…) because I can't think of any words to (…)

*Interpreter*: Nothing comes to mind.

*Researcher and interpreter*: ((laughing))

*Researcher*: Well something comes to mind, that you don't understand (…) something, doesn't it?

*Boy*: Well (…) you, you can get depressed for a reason, I don't know, I've never had those kinds of changes, I don't know.

The mental health association game was envisioned as a break from the more formal questions. However, by asking them to say whatever came to mind based on mental health-related words chosen by the researchers, it is possible that some of the youth considered it more like a test. Prompting the participants to speak freely may have been an unfamiliar or uncomfortable way for some of the youth to discuss mental health.

In addition, there may have been some confusion regarding the role of the researcher and their motivation or reasons for coming there to speak with them. Some considered the interview a kind of screening and one boy jokingly asked us:

*Boy*: Finished? So do I need a psychologist?

*Researcher*: Sorry?

*Boy*: I need a doctor?

The instructors facilitating the YAM sessions and mental health researchers from the University were often considered one and the same: “they came up from x University to give us talks and that”, and were thus perhaps thought of as authority figures.

Many of the youth struggled to find words that they deemed acceptable to use when speaking to mental health professionals. Usually stress came to mind as an overarching word to describe discomfort or mental ill health. One girl explained, “Stress and depression are mainly the same.” Stress was also used to compare and contrast with other words and feelings. Consider the following example a boy shared with us:

A moral crisis is losing something. For example a friend goes abroad like my friend went to live in England. For example this is a loss for me and crisis to me means losing something. I wasn’t stressed, I can’t call it stress, it is just crisis. Crisis to me is like losing something.

The closer we listened, after setting aside psychiatric and psychological classifications, and indeed sometimes only at the time of the analysis, the more the youth were able to tell us, in their own words, about mental health and YAM. One girl described mental health to us in a particularly poignant fashion:

Mental is like (…) I can´t explain, physical is what you can touch and feel but mental is thoughts and internal things.

**We are all human, duh!** Taking another look at Fig 1, the word *Duh*! can be found floating between the three columns. *Duh* is an American expression popular from the 1980s to this day, commonly used sarcastically by youth when someone states the obvious. It can be used to express actual or feigned ignorance or stupidity [50]. Here *Duh*! hovering in the background is used to illustrate the reaction of many of the youth when we asked questions about mental health. *Duh*! is in response to the tendency of researchers to sometimes ask questions bordering on the obvious, at times acting as if youth know less than adults about their own mental health-related experiences. Even if the youth did not express this by using *duh* specifically, the sentiment was noticeable in the interviews, both verbally and non-verbally.

*Researcher*: Do you think young people have a lot of problems like that, like we had in this proje…

*Girl*: Yeah

*Researcher*: Yeah? Which ones are those?

*Girl*: Most of them. Well, the thing with the vices, drugs. How can I say, with alcohol, with depression, with broken hearts, a lot (…) But, mostly all, and the ones with the parents. So, everything, others (…)

*Researcher*: Everything? It’s…

*Girl*: ((laughing)) It’s normal, this is why the course [YAM] was made, right?

*Interpreter*: What?

*Girl*: I’m saying that this is the point, because this is the purpose of the course, right?

The girl here tells us, somewhat sarcastically, that obviously young people have mental health issues and that we know so much or YAM would not exist in the first place. *Duh*! epitomizes the importance of not minimizing the experience and opinions of youth and, instead, to take the time to listen to their accounts. This is especially important when considering young people’s tendency to express themselves beyond professionalized mental health vocabulary.

If the YAM instructors and the researchers were sometimes thought to be judging the youth, then teachers were even guiltier of such behavior. Not talking to teachers or other school personnel about mental health, was in part mentioned as having to do with them evaluating and grading them, but also because the occasion rarely presented itself. Many of the youth did however mention discussing mental health with their peers.

*Interpreter*: Does it usually happen to you to have discussions like these in classroom? Do you talk about it?

*Boy*: Hmmm, no. Because I'm not (…) I mean, it depends, among us, among us classmates, but with the teachers it is not very common.

*Interpreter*: And among yourselves, what do you talk about?

*Boy*: Oh well, mostly, I don’t know, if it come out during a discussion, I don’t know, a guy who smokes, stuff like that. We talk, I mean, I give my opinion, I ask "why are you doing this?” at the end it’s something that hurts you, "why do you have to do it?" and so there is a debate. Also because in the end we don’t all think in the same way.

Comparing YAM to other health promotion or prevention programs, this girl told us:

*Interpreter*: Had you ever taken part in similar programs at school?

*Girl*: Programs like this about bullying and that, no. On drugs and alcohol and that, yes.

*Researcher*: What were those programs like? Were there talks about (…)?

*Girl*: Yes, there were talks, they came to speak to us and then they gave us exercises to do and we had debates in class. Or they divided the class in two groups and you had to debate against the others.

*Interpreter*: And if you compare the programs (…) how were they different or better?

*Girl*: Well, the others were more boring because they gave talks and you had to put up with it, you know, a pain actually. [In YAM] they talked and that, but for example we did the performances [role-plays]. Anyway, it was alright.

### Distribution of youth

This study did not aim to explore gender differences, yet it can be noted that there are some trends. The distribution of the interviewed youth into the five groups was quite even in terms of numbers and country where they lived, but only males were identified as belonging to the “not my topic” group and more females were in the “careful and “respect for authority” groups.

## Discussion

### YAM, youth in the present

To reduce mental health stigma, it has been observed that it is useful to start with “what matters most” to a particular community [30]. YAM consequently aims for a less anticipatory approach to mental health by providing youth with a safe space for play and discussion. Scientific assessments, probable outcomes or professionalized language are not pitted against the everyday meanings that youth give to their experiences. Rather, every YAM group is invited to make the program their own. Reflection stands at the core as the youth discuss and compare their different perspectives and think about how to care for themselves and support each other.

Each group of youth influences YAM in a slightly different direction every time the program is implemented. The classroom environment, upcoming school-related events, what is going on the personal lives of the youth present, peer group dynamics and numerous factors beyond the scope of YAM may influence the course of YAM. Similarly, the participants’ interest in and experiences of mental health and their interactions with mental health professionals matter, both as individuals and as a group. In any given YAM context, youth will engage with the program in many different ways. Some will favor participating actively while others will observe the role-plays; some will discuss and try out different solutions as a group, while others might prefer not to engage in the debates; some will leaf through or read the booklet and might keep it at home for later reference, while others may turn to the instructors for advice.

Some youth have more training in using mental health jargon or interest in speaking with mental health professionals, while others find such exchanges less appealing or almost intolerable. Mistrust of, or bad experiences with mental health professionals, ease and willingness to speak with adults, shame, and other motivations all play part in how youth engage with mental health interventions. These interviews did not provide an opportunity to better understand the underlying reasons for such tendencies; however, we were able to detect and identify different strategies and positions the youth held towards the researchers. We also observed that the researchers brought their own expectations that led to anticipating answers, stating the obvious, or getting along better with the youth who expressed themselves more freely. Paying attention and carefully describing the dynamics between researchers and youth can be helpful for exploring how youth relate to all kinds of encounters with mental health professionals, including YAM instructors.

Like any mental health promotion, YAM appeals to some more than others in its intended audience. The “engaged” group tends to be both seen and heard in the classroom and alongside “eager to please”, and they are perhaps the most likely to actively engage with the instructor. The “disengaged” ones can be found at the other side of the spectrum, disinterested in active participation. However, those in the “initially hesitant” or “cautious” groups also took active part in YAM, though perhaps less conspicuously than the other groups. Beyond observations relating to more or less active participation in the YAM classroom, these interviews provide some nuance to the variation in experience for the youth participating in the program.

### Moving toward the youth in youth aware of mental health

YAM actively invites youth to lead role-plays and conversations about emotions, compassion and solidarity, yet, the simple fact that mental health is on the table alienates some youth. Youth in the “disengaged” group spoke of disinterest and critiqued the methods but also evoked shame, inhibition, and intimidation. Those not interested in mental health, those who believed such matters should not be discussed publically and that seeking help was not desirable, and those who lacked the vocabulary to do so were the most difficult to reach with a program like YAM. Perhaps YAM in its current form did not provide a comfortable place for them. Yet, it’s worth noting, they were also willing to participate if it meant avoiding regular classes.

The “cautious” group sometimes struggled to come to terms with what was going on in the classroom with all the commotion surrounding the role-plays and discussions. They did not appreciate speaking in front of the class, but by observing others role-play, reading the booklet and reflecting on their own, they identified ways of dealing with the topics at hand. The “cautious” group objected to some methods such as exploring topics as a group and discussing them in public, but acknowledged that YAM allowed them to communicate without telling their specific stories. They were a bit concerned with the hands-off style of the YAM instructors and may have been more accustomed to a more directive teaching style.

The “cautious” and “disengaged” groups did not always recognize the purpose of YAM or agree with all the program methods, and in future mental health promotion programs it would be important to take this into consideration. Perhaps adding a component that specifically calls for silent or written reflections would appeal to some of the youth who felt alienated by active classroom participation.

The youth in the “initially hesitant” group especially took to the role-plays and debates. This was mainly because these activities invited personal opinion and experience, avoided simplifications of complex topics, were easy to relate to, and fun. They appreciated the real life applicability of the concepts in YAM such as understanding the consequences of your actions and acknowledging the existence of a variety of perspectives on mental health. Hearing their peers share opinions on topics not typically discussed in the classroom was significant to them.

The “eager to please” group emphasized their reverence for mental health professionals, both the YAM instructors and those listed in the community-based resources. Despite their respect for professionals, some felt “watched” during YAM and many of them spoke of the importance of picking yourself up and moving on without the help of others. To some extent this approach is contrary to the solidarity that transpires in YAM, but be that as it may it is an important perspective to discuss during the program. Yearning to grow up, they voiced the wish that their peers would do so as well.

The “cautious”, “initially hesitant” and “eager to please” groups overlapped in certain regards such as showing some apprehension or reluctance to actively participate. Yet, over time, they found that particular aspects of YAM worked for them. Perhaps they valued quietly watching others, discussing or even being in disagreement with their peers on some subjects, while others found encouragement in the resource list provided.

The “engaged” group valued a mental health dialogue even before introduction to YAM and appreciated the reinforcement the program provided. Enthusiastically participating, they echoed many of the YAM objectives such as supporting their peers and reflecting on how one’s actions influences how one feels. Perhaps the YAM instructors tended to lean on these youth, as they were typically the first to participate in the role-plays. Their insights were not necessarily related to personal experiences but were often expressed as beyond the self, with subtle regard to the multitude of expressions of mental health. They, like the “initially hesitant” group, enjoyed hearing other classmates speak and both of these groups appreciated the autonomy the instructors granted them.

Youth from the “engaged”, “initially hesitant” and even “eager to please” groups articulated a reinforced sense of knowledge about mental health and increased capacities for problem solving after YAM. They mentioned reflecting on the program contents over time, using tools they had learned in the program to help their friends, and even gave specific examples of situations in which they had returned to YAM know-how before acting. They found YAM useful because the role-plays prompted them to use examples from their everyday lives. Discussions in the classroom led them to learn of the different opinions of their classmates, and sometimes the discussions continued beyond the scope of the program.

### YAM, a universal program for youth

YAM is a universal mental health promotion program, welcoming youth of all backgrounds and experiences, invariably involving diverse groups of youth with varying levels of comfort around mental health topics. Some of the youth will not necessarily be interested in mental health topics, will not actively participate in the discussions or role-play, or may feel relatively uneasy with mental health professionals. As expressed in a previous article by the authors, the heterogeneity of the participants can in fact be one of the reasons for the program’s success [29], more specifically by encouraging a variety of perspectives.

Although there is certainly some overlap between the five categories of youth, paying attention to the main distinctive features between the groups can help us to improve future mental health promotion. Awareness of the diversity present in every group of youth on the part of program creators and instructors means paying attention to the levels of interest and desire to participate as well as noting comfort in interacting with mental health professionals. In addition, an accessible environment for youth with a variety of learning styles should be provided. The topics discussed in YAM are not necessarily easy to think and speak about, especially if the youth present are engaging with said topics in public for the first time. It is up to YAM to make sure that sufficient support is provided to the participants by helping to make the role-plays and discussions safe and intelligible. One of the “engaged” girls quoted in the Results section expressed it quite clearly:

It’s hard to find your voice to fully express them [emotions]. I think that.

### Researchers and youth: A question of power dynamics

As researchers, answering to both the scientific community and those being studied is imperative. Holding honest and open dialogues about the experiences of youth is contingent on a lot of factors, and we here focus on the modes of interaction of mental health researchers as well as the positioning of youth in conversation with us. While unpacking the relationship between youth and researcher, we have not pronounced any of the two parties as initiator of said dynamics, but instead concentrated on the reciprocity of the interaction. However, the warm atmosphere of the qualitative interview belies the fact that most of the power lies in the hands of the researcher who sets the agenda, controls the content, and decides when to terminate the conversation [36, 49].

Despite clear indications in the interview guide to be good listeners and to refrain from dominating the conversation, the researchers sometimes strayed from the guidelines and the interviews contain noticeable power differences. In effect, the very nature of such interviews invites some of these power differences, especially since the researchers control not only who will be the target group, but also the content of the conversations. Although we did not set out to engage with youth in a manipulative way, some of the quotations in the Result section reveal such dynamics.

Professional and personal assumptions about youth, mental health, and YAM informed the interviews and at times the bias was more explicit, as in the case of leading questions, not relinquishing previous definitions or when disagreeing with the youth. Leading questions are related to different inclinations, such as the wish to confirm a hypothesis, get closer to or perhaps overestimate one’s understanding of the person interviewed based on assumptions or a particular world view. Confirmation bias can be thought of as a way to filter and understand information. On the other hand, the youth have the agency to decide whether or not to attend, answer to the questions posed and to respond as they see fit. Even in the case of leading questions, the youth can always interpret and respond in a possibly generative direction, perhaps even different from what the researcher intended. In interviews when the researchers realized that they were steering the conversations or acting in a patronizing way, they usually tried to stop themselves. However, conceivably the damage was already done.

Some of the youth mentioned the feeling of being watched over or that they thought of the interview as a mental health screening. This may point to the infrequency of adults engaging with youth in dialogue about mental health. In future initiatives, it will be important to properly address this aspect in order for youth to feel at ease. It has by no means been an easy process to put our bias on display. However, we believe that we can learn for future work of this kind and hopefully inspire others to inspect their interactions with youth more closely.

#### The gap between language and experience

A point of contention observed in the interviews was that of contrasting language between youth and researchers. This was noted in moments of silence, disagreement, expectation, prompting and when some of the youth in the “careful”, “not my topic” and “respect for authority” groups struggled to use the terms proposed by the researchers. When the researchers were unfamiliar with certain expressions or were faced with reticence or youth who would speak few words, the interaction between the two parties was affected. At times, despite aspirations on the part of the researcher to hold an open dialogue, previously held ideas and definitions could not be relinquished. We labeled these tendencies as “frustrated”, “out of sync” and “insecure” on the part of the researchers. The researchers also identified favorites in the “interested” and “foot in the door” groups with whom the conversation flowed without much effort and the interview approach was here that of “favoritism” and “familiarity”.

Similar dynamics likely transpire in other encounters between youth and researchers, including mental health interventions such as YAM. While the task of psychiatric language is to lift experiences out of sociocultural and historical contexts to the generalizable, we cannot forget the origin of these classifications: human experience. In dialogue, and particularly by listening to youth, we can find a way back to those experiences. After all, mental health, as named and categorized by professionals, is not universally recognized.

#### Having fun matters

Adults often treat youth as if they know more than them. Youth are not indifferent to this tendency, and react accordingly. The resulting dynamic is not conducive to good mental health promotion. As expressed by one of the participants, mental health promotion cannot simply be “cheesy self-help”.

A motivating force for youth to participate in YAM as well as the interview was simply to do something different or more fun than regular classes. Youth may be more willing to participate in mental health initiatives, and perhaps even actively, if they enjoy themselves or if the activity is “a bit of a laugh”. Laughter can invite the nuance difficult topics require. Youth from all groups who participated in YAM recalled funny or norm-breaking role-plays, such as those where the genders were switched or someone acted the role of an adult. In the interviews, subtle jokes were used to ease discussion about more sensitive topics. Jokes and laughter serve numerous roles: to help overcome language difficulties, agree on something, leave a difficult topic, enter a lighter one and, in some instances, to digest sensitive information.

### Distribution of youth: A reality more complex than the data

This study did not aim to explore gender differences, particularly since it is a small study sample, yet it can be noted that there are some trends in the distribution, with exclusively males in the “disengaged” group and more females in the “cautious” and “eager to please” categories. It should be noted that all of the researchers and three out of four interpreters were female.

It is possible that some boys, like the ones in the “disengaged” group, were disinterested in programs about mental health in general. This may have been especially the case when it involved voicing your own opinions or sharing your experiences in front of peers and mental health professionals. It would be important to understand how better to involve this group. The “cautious” group, which had more females than males, reminds us to create programs that nurture safe spaces for youth that may not readily or actively participate in debates and role-plays about mental health. Neither of these two groups wanted to share personal stories. Both these groups of youth probably require long-term relationships to discuss mental health with professionals and future research should contain more than one interview meeting and perhaps invite youth to participate in focus groups to not have to express their views alone with adults.

The “cautious” group, may not have been entirely comfortable with the conduct of the instructor while the “eager to please” group may have felt apprehensive of support from their peers. These positions may compel future YAM facilitators to be more transparent about the reasoning behind a more permissive teaching style and inviting youth to take charge and educate each other. Such adjustments would benefit all participating youth and allow for the cultivation of a safe space.

### Limitations and future research

The majority of the interviews were conducted with an interpreter. This may have led to some confusion and at times hindered more animated discussions. The switch of languages between English and the native tongue of the youth may have been challenging for some.

Moreover, the interviews were held in schools, which inherently pose several limitations. Historically, schools are a place for reformation and preparation, for training youth to perform [51]. It is possible that some youth consider mental health professionals as part of the school environment and as associated with teachers and the curriculum, conceivably there to evaluate them. In future work, it would be important to critically understand the role of the school context, as it is the environment of many public mental health interventions.

Due to sample size, the data collected do not allow us to speculate further about the role of gender or explore the many structural, cultural, and political aspects of youth, such as race, gender identity beyond the female/male dichotomy, ethnicity, social class, sexual orientation, disability, or any other markers of difference. Moreover, the study of cultural practices of peer culture and cliques in the school context would help us better understand YAM participation. All the above-mentioned markers may directly influence the youth’s expectations, desires and thoughts about mental health, experience of mental health professionals, and participation in mental health promotion. Of course, such variables do not only influence the lives of youth, but those of researchers as well, an aspect observed and evaluated in the course of the self-reflexive data analysis. Importantly, the researchers’ values played a role not only in creating the protocol or conducting the interviews, as seen particularly in the case of leading questions, but irrefutably in the interpretation of the data as well. The positioning of the researcher in relation to the youth certainly influenced the results here presented. However, in exploratory research we are not after one given truth. Instead, all knowledge is considered as partial and an interpretation of reality.

Further study on all these subjects is needed to better understand how groups of youth engage with mental health promotion and how youth and researchers interact. Participant observations during mental health promotion initiatives, individual interviews, as well as focus groups with youth are conducive to deeper understanding of such topics. Importantly, we believe that in addition to studying and listening, actual collaborations with youth to create future interventions are essential.

## Conclusions: Listen closely and be cognizant of power dynamics

In the evaluation of mental health promotion, considerable attention is given to health and behavioral outcomes. More textured accounts of experience are rarely published and research that includes reflection on the language and biases of professionals is rare. Perhaps the main reason for this is simply a lack of precedence. Qualitative research can be of help in orienting the conversation towards topics that matter to youth. By listening closely, we can find a way to their experiences. However, it is imperative to approach such dialogue with care, conscious of the power dynamic present in the encounter between mental health professionals and youth. Future initiatives should remain cognizant of the expectations of researchers and their possible tendencies to favor certain youth while perhaps inadvertently alienating others.

Speaking about mental health is not necessarily effortless or appealing–for anyone. This study helps us to recognize youth who “don’t know what to do with these words”, those who observe quietly without actively engaging, those who prefer to speak from personal experience and leave generalities behind, those who believe we need to solve our problems on our own, alongside those who enjoy exploring mental health topics. Youth of all backgrounds should be asked to collaborate with professionals in the creation of future mental health promotion, but let’s not stop there; in such efforts, we need to be attentive to the intentions and modes of interaction of mental health professionals.

## Supporting information

S1 File Interview guide. (PDF)

## References

[1] Mawn L, Welsh P, Stain HJ, Windebank P. Youth Speak: increasing engagement of young people in mental health research. J Ment Health. 2015;24:271–275. doi: 10.3109/09638237.2014.998810 26193175 10.3109/09638237.2014.998810

[2] Tilleczek KC. Approaching youth studies: being, becoming, and belonging. Don Mills, Ont.; New York: Oxford University Press; 2011:xii, 163.

[3] Bryman A. Social research methods. Oxford university press; 2012:178–179;573–575.

[4] Ibrahim A. Critical Youth Studies: An introduction In: Ibrahim A, Steinberg SR, editors. Critical Youth Studies Reader. Peter Lang Publishing Inc.; 2014 p. xv–xix.

[5] Sawyer SM, Afifi RA, Bearinger LH, Blakemore S-J, Dick B, Ezeh AC et al Adolescence: a foundation for future health. The Lancet. 2012;379:1630–1640. 10.1016/S0140-6736(12)60072-5 22538178

[6] Gunnell D. A Population Health Perspective on Suicide Research and Prevention. Crisis. 2015;36:155–160. doi: 10.1027/0227-5910/a000317 26266821 10.1027/0227-5910/a000317

[7] Hjelmeland H, Knizek BL. Why we need qualitative research in suicidology. Suicide Life Threat Behav. 2010;40:74–80. doi: 10.1521/suli.2010.40.1.74 20170263 10.1521/suli.2010.40.1.74

[8] Greenhalgh T, Annandale E, Ashcroft R, Barlow J, Black N, Bleakley A et al An open letter to The BMJ editors on qualitative research. BMJ. 2016;352:i563 doi: 10.1136/bmj.i563 26865572 10.1136/bmj.i563

[9] Christensen PH. Children’s participation in ethnographic research: issues of power and representation. Children & society 2004

[10] Lesko N, Talburt S. Keywords in youth studies: tracing affects, movements, knowledges. New York: Routledge; 2012:ix, 344.

[11] Hansen H, Dugan TM, Becker AE, Lewis-Fernández R, Lu FG, Oquendo MA et al Educating psychiatry residents about cultural aspects of care: a qualitative study of approaches used by U.S. expert faculty. Acad Psychiatry. 2013;37:412–416. doi: 10.1176/appi.ap.12080141 24185288 10.1007/BF03340081

[12] Wasserman D, Carli V, Wasserman C, Apter A, Balazs J, Bobes J et al Saving and empowering young lives in Europe (SEYLE): a randomized controlled trial. BMC Public Health. 2010;10:192 doi: 10.1186/1471-2458-10-192 20388196 10.1186/1471-2458-10-192 PMC2880291

[13] Carli V, Wasserman C, Wasserman D, Sarchiapone M, Apter A, Balazs J et al The saving and empowering young lives in Europe (SEYLE) randomized controlled trial (RCT): methodological issues and participant characteristics. BMC Public Health. 2013;13:479 doi: 10.1186/1471-2458-13-479 23679917 10.1186/1471-2458-13-479 PMC3665603

[14] Report WE-STAY. WE-STAY Report (2013). Final Report to the EU Commission. Available for download at www.we-stay.eu/WESTAY_finalreport.pdf. 2013

[15] Wasserman D, Hoven CW, Wasserman C, Wall M, Eisenberg R, Hadlaczky G et al School-based suicide prevention programmes: the SEYLE cluster-randomised, controlled trial. Lancet. 2015;385:1536–1544. doi: 10.1016/S0140-6736(14)61213-7 25579833 10.1016/S0140-6736(14)61213-7

[16] Website YAM. http://www.y-a-m.org. Last accessed on Jan 24th 2018.

[17] Brunner R, Kaess M, Parzer P, Fischer G, Carli V, Hoven CW et al Life-time prevalence and psychosocial correlates of adolescent direct self-injurious behavior: a comparative study of findings in 11 European countries. J Child Psychol Psychiatry. 2014;55:337–348. doi: 10.1111/jcpp.12166 24215434 10.1111/jcpp.12166

[18] Brunstein Klomek A, Snir A, Apter A, Carli V, Wasserman C, Hadlaczky G et al Association between victimization by bullying and direct self injurious behavior among adolescence in Europe: a ten-country study. Eur Child Adolesc Psychiatry. 2016;25:1183–1193. doi: 10.1007/s00787-016-0840-7 27010553 10.1007/s00787-016-0840-7

[19] Carli V, Hoven CW, Wasserman C, Chiesa F, Guffanti G, Sarchiapone M et al A newly identified group of adolescents at “invisible” risk for psychopathology and suicidal behavior: findings from the SEYLE study. World Psychiatry. 2014;13:78–86. doi: 10.1002/wps.20088 24497256 10.1002/wps.20088 PMC3918027

[20] Durkee T, Carli V, Floderus B, Wasserman C, Sarchiapone M, Apter A et al Pathological Internet Use and Risk-Behaviors among European Adolescents. Int J Environ Res Public Health. 2016;13 10.3390/ijerph13030294 PMC4808957 27005644

[21] Kaess M, Brunner R, Parzer P, Carli V, Apter A, Balazs JA et al Risk-behaviour screening for identifying adolescents with mental health problems in Europe. Eur Child Adolesc Psychiatry. 2014;23:611–620. doi: 10.1007/s00787-013-0490-y 24248753 10.1007/s00787-013-0490-y

[22] Nakar O, Brunner R, Schilling O, Chanen A, Fischer G, Parzer P et al Developmental trajectories of self-injurious behavior, suicidal behavior and substance misuse and their association with adolescent borderline personality pathology. J Affect Disord. 2016;197:231–238. doi: 10.1016/j.jad.2016.03.029 26995466 10.1016/j.jad.2016.03.029

[23] Kaess M, Parzer P, Brunner R, Koenig J, Durkee T, Carli V et al Pathological Internet Use Is on the Rise Among European Adolescents. J Adolesc Health. 2016;59:236–239. doi: 10.1016/j.jadohealth.2016.04.009 27267140 10.1016/j.jadohealth.2016.04.009

[24] McMahon EM, Corcoran P, O’Regan G, Keeley H, Cannon M, Carli V et al Physical activity in European adolescents and associations with anxiety, depression and well-being. Eur Child Adolesc Psychiatry. 2017;26:111–122. doi: 10.1007/s00787-016-0875-9 27277894 10.1007/s00787-016-0875-9

[25] Wartberg L, Brunner R, Kriston L, Durkee T, Parzer P, Fischer-Waldschmidt G et al Psychopathological factors associated with problematic alcohol and problematic Internet use in a sample of adolescents in Germany. Psychiatry Res. 2016;240:272–277. doi: 10.1016/j.psychres.2016.04.057 27138817 10.1016/j.psychres.2016.04.057

[26] Banzer R, Haring C, Buchheim A, Oehler S, Carli V, Wasserman C et al Factors associated with different smoking status in European adolescents: results of the SEYLE study. Eur Child Adolesc Psychiatry. 2017;26(11):1319:1329 doi: 10.1007/s00787-017-0980-4 28386649 10.1007/s00787-017-0980-4 PMC5656692

[27] Koenig J, Brunner R, Fischer-Waldschmidt G, Parzer P, Plener PL, Park J et al Prospective risk for suicidal thoughts and behaviour in adolescents with onset, maintenance or cessation of direct self-injurious behaviour. Eur Child Adolesc Psychiatry. 2017;26:345–354. doi: 10.1007/s00787-016-0896-4 27558490 10.1007/s00787-016-0896-4

[28] Bousoño Serrano M, Al-Halabí S, Burón P, Garrido M, Díaz-Mesa EM, Galván G et al Substance use or abuse, internet use, psychopathology and suicidal ideation in adolescents. Adicciones. 2017;29:97–104. doi: 10.20882/adicciones.811 28170053 10.20882/adicciones.811

[29] Wasserman C, Hoven CW, Wasserman D, Carli V, Sarchiapone M, Al-Halabí S et al Suicide prevention for youth—a mental health awareness program: lessons learned from the Saving and Empowering Young Lives in Europe (SEYLE) intervention study. BMC Public Health. 2012;12:776 doi: 10.1186/1471-2458-12-776 22971152 10.1186/1471-2458-12-776 PMC3584983

[30] Susser E, Patel V. Psychiatric epidemiology and global mental health: joining forces. International journal of epidemiology. 2014 1–7. 10.1093/ije/dyu053 24659583

[31] Snaza N, Weaver JA. Posthuman(ist) Youth: Control, Play, and Possibilities In: Ibrahim A, Steinberg S, editors. Critical Youth Studies Reader 2014 p. 349–359.

[32] Boler M. Feeling power: emotions and education. New York: Routledge; 1999:xxix, 235.

[33] Guest G, Bunce A, Johnson L. How many interviews are enough? An experiment with data saturation and variability. Field methods. 2006;18:59–82.

[34] Morse JM. Determining sample size. Qualitative health research. 2000;10:3–5.

[35] Sofaer S. Qualitative research methods. International Journal for Quality in Health Care. 2002;14:329–336. 12201192 10.1093/intqhc/14.4.329

[36] Kvale S. The interview situation. Interviews: An Introduction to Qualitative Research Interviewing. 1996 p. 124–143.

[37] Clemence A, Doise W, Lorenzi-Cioldi F. The quantitative analysis of social representations. Routledge; 2014

[38] Dedoose. Web application for managing, analyzing, and presenting qualitative and mixed method research data. Los Angeles, CA: SocioCultural Research Consultants, LLC (www.dedoose.com). 2014

[39] Charmaz K. Qualitative interviewing and grounded theory analysis In: Gubrium JF, Holstein JA, editors. Handbook of interview research: context and method. Thousand Oaks: Sage; 2004.

[40] Rapley TJ. The art (fulness) of open-ended interviewing: some considerations on analysing interviews. Qualitative research. 2001;1:303–323.

[41] Davies CA. Reflexive Ethnography: A Guide to Researching Selves and Others (The ASA Research Methods). Routledge; 2007:320.

[42] Coffey A, Atkinson P. Making sense of qualitative data: complementary research strategies. Sage Publications, Inc; 1996

[43] Cohen DJ, Crabtree BF. Evaluative criteria for qualitative research in health care: controversies and recommendations. Ann Fam Med. 2008;6:331–339. doi: 10.1370/afm.818 18626033 10.1370/afm.818 PMC2478498

[44] Tong A, Sainsbury P, Craig J. Consolidated criteria for reporting qualitative research (COREQ): a 32-item checklist for interviews and focus groups. Int J Qual Health Care. 2007;19:349–357. doi: 10.1093/intqhc/mzm042 17872937 10.1093/intqhc/mzm042

[45] Yardley L. Dilemmas in qualitative health research. Psychology and health. 2000;15:215–228.

[46] Forbat L, Henderson J. Theoretical and practical reflections on sharing transcripts with participants. Qual Health Res. 2005;15:1114–1128. doi: 10.1177/1049732305279065 16221883 10.1177/1049732305279065

[47] Ponterotto JG. Brief note on the origins, evolution, and meaning of the qualitative research concept thick description. The Qualitative Report. 2006;11:538–549.

[48] Geertz C. The interpretation of cultures: Selected essays. Basic books; 1973

[49] Brinkmann S, Kvale S. Confronting the ethics of qualitative research. Journal of constructivist psychology. 2005

[50] Website M-W. http://www.merriam-webster.com/dictionary/duh. Last accessed on Jan 24th 2018.

[51] Martin E. Imagining mood disorders as a public health crisis In: Serlin D, editor. Imagining illness: public health and visual culture. Minneapolis, MN: University of Minnesota Press; 2010 p. 245–263.

