# Supplementary material for: Interactions between youth and mental health professionals: The Youth Aware of Mental health (YAM) program experience
Source: PLoS One. 2018 Feb 8;13(2):e0191843. doi: 10.1371/journal.pone.0191843 (PMC5805239; doi:10.1371/journal.pone.0191843)
Supplement: S1 File — (PDF) [file pone.0191843.s001.pdf]

Interview guide

Greetings

Icebreaker

I. YAM/AWARENESS PROGRAM

REMEMBERING THE STUDY

YAM/AWARENESS PROGRAM

YAM – INTRO

WORKSHOPS/ROLE-PLAY

INSTRUCTOR

BOOKLET

EVALUATION OF THE PROGRAM

II. MENTAL HEALTH UNDERSTANDING

MENTAL HEALTH – INTRO

EMOTIONAL AND RELATED “PROBLEMS” OF ADULTS & YOUTH

HELP

III. SKIPPING SCHOOL

SKIPPING SCHOOL - THE WHO, WHY AND HOW

CLASSMATES

SCHOOL AND TEACHERS - AWARENESS, CONSEQUENCES ...

PARENTS AND SKIPPING SCHOOL

YOUR EXPERIENCE

QUESTIONNAIRE

UNDERSTANDING OF QUESTIONNAIRE

SPECIFIC QUESTIONS REGARDING THE QUESTIONNAIRE, IN CASE THERE IS TIME

CLOSING

Semi-structured interview with YAM participants

START RECORDING as soon as possible after the person being interviewed agrees to it (they were all informed about it prior to agreeing to take part; simply inform them about the content and reason for the interview again to get additional oral consent).

Greetings

*"We are interested in hearing your opinions **outside of the very strict questionnaire** format where you fill in boxes and to talk to you more freely about some of the questions and issues that were raised in the recent YAM/WE-STAY program in your school."*

Tell the student about local interviewer and Vita/Camilla. Where we are from, that we like to talk to the students directly to hear their opinions, we have done interviews like this in the past, that we will talk to students in four European countries, their age... By listening to the students directly we hope to make better programs for youth in the future.

Before we start we also say

*"Everything you say to us will **remain confidential** and we will not tell anyone about what you are telling us, only in case something you say shows that you are in danger of harm in some way. We will ask you some questions and if you do not want to answer some of the questions you don't have to tell us or even tell us why you don't want to tell us." "We would like to **record** this conversation so that we can go back to it later and listen to your answers, especially since I don't speak your language – it will be very helpful to have the conversation on an audio recording to later better understand what was said. Is this ok with you? Of course only the research team will listen to this recording and no one else like your teachers or anyone from school. Often when you do research projects like this to understands adolescents, it is not easy to get a feeling for what they actually think if they are simply ticking off boxes or answering yes or no to questions, by talking to you directly we hope to better understand how you feel about the issues that we raised in our program. There are no right or wrong answers, we are just interested in your opinions, because we wish to understand young people a bit better."*

Questions and themes: the actual questions are general guidelines of what will be asked, but all the themes below should be covered in the interview. Words in parenthesis are to be used as prompts if the student does not remember or has difficulty to speak).

ICEBREAKER

Ask student what they were told about this interview.

Ask about their school year, current grade/class, their age and how long it was since YAM/WE STAY (if they remember) and then go to question number 1.

I. YAM/AWARENESS PROGRAM

REMEMBERING THE STUDY

1. Off the top of your head, what do you remember from the WE-STAY study?
2. (Questionnaire, YAM/Awareness program, what specifically from the program, the instructor, the atmosphere in the classroom, etc).

When they mention the YAM/Awareness program (role-plays/workshop) we ask them, or if they do not mention the YAM/Awareness program, then we remind them and ask them the following question:

3. What would you say YAM/the Awareness program was about?

Note the language they use for Mental Health issues and continue using it in the rest of the interview.

YAM – INTRO

4. We talked a bit about YAM/the Awareness program earlier, let's continue a bit with that, please just tell us what you **remember**, and if you don't remember something that is also ok.
    a. Was there something you did not understand?
    b. Liked? Did not like?
5. How well do you remember the YAM/Awareness Program in the WE-STAY project on a scale with 1 meaning you remember it very poorly and 7 that you remember it very well.

Very poorly	1	2	3	4	5	6	7	Very well

The questions below about the workshops, instructor and booklet are quite specific, but we expect that we will not need to ask them all in this way, since they may be covered by just allowing the student to talk freely. This section should be covered quite quickly because of time restraints. If the student does not seem to remember much, do not probe too much, simply skip to the next section.

WORKSHOPS/ROLE-PLAY

6. Tell us about the workshops. Is there anything specific you remember?
7. What did you think of the opening lecture?
8. What did you think of the role-play?
9. How much did you **enjoy** the role-play on a scale with 1 meaning you did not enjoy it at all and and 7 that you enjoyed it very much?

No, not at all	1	2	3	4	5	6	7	Yes, very much

10. Which topics in the role-play sessions do you remember? Give examples (help the student out if needed).
11. Can you tell us a bit how the role-play sessions were in your classroom?
12. Was this the first time you did role-play like this? Was it difficult?
13. Was it embarrassing to do the role-plays? Fun?
14. Did you also have discussions about the role-play? What did you say in these discussions?
15. What did you think of the closing lecture?

INSTRUCTOR

(Take note if the interviewer was the instructor)

16. What did you think of the YAM/Awareness instructor?
17. Did the instructor explain everything to you in a good way? Did you feel comfortable, safe? Was she/he fun, nice, easy to get along with?

BOOKLET

Show the booklet to the pupil to help them remember.

18. Did you **read** the booklet that was given to you? Was it easy/difficult to read?
19. How much of the "Awareness Booklet" did you read on a scale with 1 meaning you didn't read it at all and 7 that you read everything.

I didn't read anything	1	2	3	4	5	6	7	I read everything

20. Did you like the way the booklet looked?
21. After the end of the program, did you look at the booklet again?
22. Do you know where the booklet is now? (Did you throw it out?)

EVALUATION OF THE PROGRAM

23. Did you know about the **list of contacts** to health professionals at the end of the booklet? Was that list helpful in any way?

Show the contacts at the end of the booklet hand the booklet to the pupil so that she/he can look on her/his own. (

24. Did you think about contacting any of these professionals for help? (in case of need...)
25. Did anyone you know contact these professionals for help?
26. Thinking about the entire program, with the seminars, role-play, booklet, what **would you want to be different** if you did it for the first time? Would you like more or less of something? Any other topics to be covered?
27. Use your own words to describe how you found the program (interesting, helpful, not relevant, not important, boring, difficult)?
28. Was the program too short or too long?
29. Have you participated in any other similar programs in your school, which is not the Awareness Program, that is about well-being and emotions?

II. MENTAL HEALTH UNDERSTANDING

We might go into some examples that they list in this part of interview. That is not specified, but the aim is to get stories of mental health issues they came across. The timing might be a bit longer in that case.

MENTAL HEALTH – INTRO

30. Association "Game" - the words will be on sheets of paper showed to the student.

With each word, if possible ask a few follow-up questions.

"We are going to show you some words, please tell us anything and everything that crosses your mind when we say these words: For example "professor": "strict" "school" "every day" "easy to talk to"/"difficult to talk to" "different every year" "too many different professors" "classroom" "summer vacation", etc etc. Please talk as much as you can as anything you have to say is interesting to us. Do you understand? "

1. Friendship
2. Stress
3. Shyness
4. Crisis
5. Self-esteem
6. Relationship with girl or boy
7. Bullying
8. Emotions
9. Loneliness
10. Drinking alcohol
11. Taking drugs
12. Relationship with parents
13. Relationship with siblings/other family members
14. Depression
15. Mental Health
16. Broken heart
17. Psychologist
18. Feeling sad

When they finish the "flow of consciousness" we ask them: Would you use this word yourself? What else would you use if not? Would you use any of these words to describe yourself? Are any of these words closer to you than others, and why?

Do you remember any of these words from the We-Stay program?

EMOTIONAL AND RELATED "PROBLEMS" OF ADULTS & YOUTH

Thank you for doing that short game, with those answers in mind, I want to ask you some more related questions.

31. What do you think about those topics - is it common for adolescents to have emotional or mental health problems?
    a. What kind of problems?
32. What about adults? Parents?

HELP

When talking about Mental Health/emotional problems, use the words the adolescent is using, maybe it's feelings, or how I feel or something else

33. Do you think that problems like these can be helped? How?
34. What do you or people you know do when you/they have a problem?

III. SKIPPING SCHOOL

SKIPPING SCHOOL - THE WHO, WHY AND HOW

The questions below about skipping school are quite specific, but we expect that we will not need to ask them all in this way, since they may be covered by just allowing the student to talk freely.

"Now we would like to talk to you a little more freely and ask your opinion about skipping school to understand the difference between schools, cities, countries. This does not have to be about you specifically, but we want to know what you think about skipping school in general. We are interested in what students themselves think about skipping school, and not just adults or teachers or researchers like us."

35. So, tell us, what comes to mind, when I say "skipping school"?
    b. What do you, the students, call "skipping school" in this school, city, region?

    c. What do you think about skipping school, is it bad, only bad, sometimes good, when?

36. Who skips school?
37. Is there a difference if you only do it a few times or more often?
38. What would you say is a lot of skipping school and what is a little?

39. Why do people skip school (different reasons)?
40. Do the people who skip school have any specific problems? (be careful not to put this as leading question)

CLASSMATES

41. Do other students know if someone is skipping school?
42. What do you/others think about classmates, who skip school?
43. Where do students go when they skip school and what do they do?

SCHOOL AND TEACHERS - AWARENESS, CONSEQUENCES ...

44. Is it difficult or hard to skip school in your school, grade, class?
45. How do teachers, the school, the headmaster think about skipping school?
46. How do they know that a student is skipping school? Do teachers always know?
47. What do they do if they know you have skipped school?
48. How do you perceive your teachers? Does your teacher have authority? No authority? (do you follow her/his rules, wishes).
49. What would make students come to or stay in school more (if you could make any change you want)?
50. Do you think skipping school has any effect on the future?
51. Do you think that people skip school as much at your school as any other school in the city, region, the country, Europe? If you don't know, what would you guess?

PARENTS AND SKIPPING SCHOOL

52. What do your parents think about skipping school?
53. Did your parents skip school?
54. Do they sometimes cover for your absenteeism by writing an excuse?
55. If you ever skipped school, did they find out? How did they find out?
56. Would you get punished if they found out you were skipping school?
57. Do you think all parents are the same when it comes to their children skipping school? If not, tell us about some different approaches that you can think of.
58. Do you think the relationship with the parents is important when it comes to skipping school? And if so, how?
59. If you have siblings, do/did they skip school?
60. Do you think a sibling skipping school influences someone to skip/not skip?

YOUR EXPERIENCE

Even if they say they have not skipped school themselves, we want to get an idea if it is difficult or hard to skip school, what if... or ask about a student that skips: which classes they skip, if it is easier to skip classes with some teachers, what these teachers are like, if it is easier to skip in a group, why the skip, etc.

61. Have you ever skipped school?
62. Do you remember the **first time** you skipped school? Can you tell us about it?
63. If you have skipped school, what did you **do**, where did you go, etc?
64. How **often** do you skip school?
65. How do you feel when/if you do it? (feelings of guilt, courageous)
66. Do you skip school **alone** or with others?
67. Do you usually skip the same **classes**?
68. Did you skip school more or less in the past?
69. Do you want to skip school more or less?
70. How different is it to skip other activities (e.g. music lessons) that are not a part of school curriculum? (easier/harder to skip?).
71. Which classes are more or less acceptable to skip?
72. Which extra-curricular activities are more or less acceptable to skip?
73. Is there anything else you can say about skipping school?

QUESTIONNAIRE

74. Do you remember if there were any questions about skipping school in the questionnaire you did for WE-STAY?
    - If you remember these questions, what did you think of them?
75. Do you remember anything else from the questionnaire that was given to you during the WE-STAY study?
76. What did you like? Not like?
77. What would you say the questionnaire was about?

UNDERSTANDING OF QUESTIONNAIRE

Bring the questionnaire with you and show it to the pupil (questions 25 – 27)

78. Do you think the questionnaire was easy to read?

79. How did you feel about completing the questionnaire that was used in the in the “We-Stay” study, on a scale with 1 meaning you did not like it at all and 7 that you liked it very much?

I didn’t like it at all	1	2	3	4	5	6	7	I liked it very much

80. Do you remember any questions that you did not understand or did not like?
81. Was the questionnaire difficult or easy to understand on a scale with 1 being not difficult at all and 7 being very difficult?

No, not at all	1	2	3	4	5	6	7	Yes, very difficult

82. Do you remember the period when you were filling out this questionnaire and what you answered about skipping school?
    b. How honestly did you answer?
83. What about the rest of the questionnaire, how did you answer the questions (did you read all the questions, did you answer honestly, randomly, did you answer some questions in a different way, why?)
84. Have you filled in questionnaires like this before in school? or elsewhere?
85. Is there any other way that you would prefer to tell us about the issues covered in the questionnaire (use the words/language the adolescent is using)?

SPECIFIC QUESTIONS REGARDING THE QUESTIONNAIRE, IN CASE THERE IS TIME

Show them the skipping school questions and get feedback about how to ask about skipping school, cut-offs etc. Go over some other questions which were difficult to interpret.

CLOSING

86. Is there something I did not ask and would be important for us to know regarding the program or youth problems or skipping school?
87. What did you think of this interview? Was it fun? Interesting? Boring? Long?
88. Anything that made you feel uncomfortable? How do you feel now?
